# Does unified allergic airway disease impact on lung function and type 2 biomarkers?

**DOI:** 10.1186/s13223-019-0388-4

**Published:** 2019-11-27

**Authors:** Chris RuiWen Kuo, Rory Chan, Brian Lipworth

**Affiliations:** Scottish Centre for Respiratory Research, Ninewells Hospital and Medical School, University of Dundee, Dundee, DD1 9SY Scotland, UK

**Keywords:** Unified allergic airway disease, Asthma, Allergic rhinitis, Asthma control questionnaire, Fractional exhaled nitric oxide, Eosinophil, Spirometry

## Abstract

The concept of the unified allergic airway disease (UAD) recognises the association between allergic inflammation in the upper and lower airways. Patients with asthma and concomitant allergic rhinitis experience more asthma-related primary and secondary care visits. We therefore aimed to determine differences in asthma control (asthma control questionnaire ACQ-6), lung function (spirometry) and T2 biomarkers (FeNO and Eos) in relation to the presence of allergic rhinitis in patients with allergic asthma. Retrospectively, we evaluated a cohort of 60 consecutive patients with persistent asthma attending our research unit for screening into clinical trials. All included subjects were receiving inhaled corticosteroids (ICS) and had a positive skin prick test (SPT) to at least one common aeroallergen to fulfil the criterion of allergic asthma. Patients with UAD had a diagnosis of allergic asthma in addition to established concomitant allergic rhinitis. T2 biomarkers were significantly higher in patients with allergic rhinitis in contrast to those without. FEV_1_ % predicted and FEF_25-75_ % predicted were also significantly lower in patients with concomitant allergic rhinitis. However, there was no difference in ACQ-6 observed between groups. In summary, patients with allergic asthma, the presence of concomitant allergic rhinitis is associated with worse lung function and higher type 2 biomarkers.

To the Editor

The concept of the unified allergic airway disease (UAD) recognises association between allergic inflammation in the upper and lower airway. This in turn led to development of guidelines looking at allergic rhinitis and its impact on asthma [1]. Patients with asthma and concomitant allergic rhinitis experience more asthma-related primary and secondary care visits [2]. Clinical trials of intranasal steroids (INS) in patients with allergic rhinitis and asthma have demonstrated improvements in bronchial hyper-responsiveness to methacholine [3] suggesting that the upper airway may contribute downstream to asthma control. Indeed treatment with INS can reduce emergency room visits and hospitalisation for asthma [4].

To our knowledge, presently there are no studies looking at asthma control, lung function and type 2 (T2) biomarkers such as fractional exhaled nitric oxide (FeNO) and blood eosinophils (Eos), which have compared allergic asthma patients with and without allergic rhinitis.

We therefore wished to see if there were differences in asthma control (as asthma control questionnaire ACQ-6), lung function (as spirometry) and T2 biomarkers (as FeNO and Eos) in relation to the presence of allergic rhinitis in patients with allergic asthma.

Retrospectively, we evaluated a cohort of 60 consecutive patients with persistent asthma meeting the criteria, who attended our research unit for screening into clinical trials. All asthmatic subjects included were receiving inhaled corticosteroids (ICS) and had a positive skin prick test (SPT) to at least one common aeroallergen in order to fulfil the criterion of allergic asthma. Patients with UAD had a diagnosis of allergic asthma in addition to an established diagnosis of concomitant allergic rhinitis and were receiving therapy with INS with or without concurrent use of oral or intranasal antihistamine. Patients without allergic rhinitis were required to have no attributable perennial or seasonal nasal symptoms. Spirometry (Micromedical, Chatham, United Kingdom) was performed in triplicate. Caldicott guardian approval was obtained to allow access to the patient identifiable National Health Service data on blood Eos, and all patients consented for their screening data to be accessed. Comparisons for each outcome between groups were made by unpaired Student’s *t* test with alpha error set at 0.05 (2-tailed). Chi square test was conducted to assess differences within each aeroallergen of SPT in between groups. The mean difference and 95% confidence intervals (CIs) for differences are given for significant comparisons.

The overall mean age was 52 years, mean forced expiratory volume in 1 s (FEV_1_) 88% predicted, mean ACQ-6 score of 1.0, and mean ICS dose (beclomethasone equivalent) of 660 µg. 30 subjects in each group were identified with a diagnosis of allergic asthma with and without allergic rhinitis. The median number of positive SPT to common aeroallergens was 2 in both groups. The percentage sensitisation to each aeroallergen comparing allergic asthma with and without allergic rhinitis respectively were as follows: grass mix 50% vs 47%, trees 13% vs 13%, weeds 10% vs 3%, house dust mite 47% vs 70%, Aspergillus fumigatus 10% vs 7%, feathers 0% vs 3%, dog 33% vs 30% and cat 40% vs 57%. There were no significant differences within each aeroallergen between groups.

The characteristics of the study subjects and significant comparisons are summarised in Table 1. In the group with allergic rhinitis, Eos mean difference 148 (CI 48–247; p = 0.005) cells/µL and FeNO 21 (CI 7–35; p = 0.004) ppb were significantly higher than the group without allergic rhinitis (Fig. 1). The spirometry measurements were also significantly lower in patients with concomitant allergic rhinitis compared to those without, with FEV_1_% predicted: − 8% (CI − 16% to − 0.17%; p = 0.045) and forced expiratory flow at 25% to 75% of forced vital capacity (FEF_25–75_% predicted): − 16% (CI − 28% to − 4%; p = 0.008) (Fig. 1). FEV_1_ in litres was also significantly lower in allergic rhinitis: − 0.44L (CI − 0.79L to − 0.09L); p = 0.016). However, there was no difference observed in ACQ-6 (p = 0.966) when comparing groups. There were no significant correlation between FEV_1_ and FEF_25–75_ vs FeNO in either group.

**Table 1:** Characteristics of the study subjects and significant comparisons

	Allergic asthma with allergic rhinitis	Allergic asthma without allergic rhinitis
Age	55	49
F/M	17/13	11/19
SPT^†^	2 (1–4)	2 (1–3)
ICS (µg)	750	580
LABA	70%	63%
LAMA	23%	10%
LTRA	43%	17%
THEO	3%	3%
OAH	53%	0
INS	100%	0
INAH	7%	0
ACQ-6	1.03 (0.13)	1.04 (0.17)
FEV_1_ (% predicted)	84 (3)	92 (3)*
FEF_25–75_ (% predicted)	43 (4)	59 (5)**
FeNO (ppb)	50 (6)	29 (4)**
Eos (cells/µL)	380 (34)	233 (36)**

ICS as beclomethasone equivalent dose

*LABA* long acting β2 agonist, *LAMA* long acting muscarinic antagonist, *LTRA* leukotriene receptor antagonist, *THEO* theophylline, *OAH* oral antihistamine, *INS* intranasal steroid, *INAH* intranasal antihistamine

Values are presented as mean (SEM), ^†^median (IQR). *p < 0.05, **p < 0.01

**Fig. 1:**
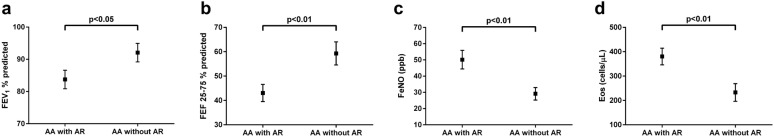
Values are shown as means and standard error of means for significant comparisons between allergic asthma with and without allergic rhinitis according to **a** FEV_1_% predicted, **b** FEF_25–75_% predicted, **c** FeNO and **d** blood eosinophils

Comparing our results to previous study, Gratziou et al. [5] demonstrated that FeNO was significantly higher in patients with concomitant allergic asthma and rhinitis compared to non-allergic patients, although their study did not differentiate with regards to allergic asthma without concomitant allergic rhinitis. Our results showed that the T2 biomarkers were higher in the group with UAD despite the concomitant use of INS and ICS, with the mean ICS dose being approximately 200 µg higher than those without allergic rhinitis. However, a previous study has shown that blood eosinophil counts were significantly suppressed from corticosteroid naïve baseline by combined treatment with INS and ICS in patients with UAD [6]. Notably we found no difference in the number of positive skin prick tests between the two groups indicating that the allergic burden was comparable. Hence, the presence of rhinitis rather than allergen sensitisation per se in UAD is the most likely explanation for the observed results. The patients with UAD were taking a 29% higher dose of ICS which presumably reflects the disease burden.

A higher T2 burden mirrored poorer lung function in the patients with UAD. In our study, the mean difference in FEV_1_ between groups exceeded the minimal clinically important difference of 230 ml. This supports the presence of cross-talk between upper and lower airway mucosa in response to allergy related T2 inflammation in UAD. High T2 biomarkers especially blood eosinophils are associated with more severe asthma [7]. In severe persistent asthma with high T2 biomarkers, biologic therapies directed at IL-5 and IL-13 result in lower circulating levels of eosinophils and immunoglobulin E, which in turn reduces asthma exacerbations [8, 9].

A previous study in children revealed that the presence of allergic rhinitis was associated with worse asthma control in terms of paediatric ACQ [10]. Conversely, our study has shown no significant difference in ACQ comparing the two groups. This may reflect the high proportion of patients on second line controller therapy in patients with UAD.

We appreciate that there are limitations to our study. Firstly, we used retrospective cross-sectional data and hence prospective evaluation might be able to further evaluate the influence of UAD and associated therapies over time. Secondly, since ACQ represents a snapshot of the previous week, it is conceivable that if prospective consecutive evaluation had been performed then we might have shown worse asthma control in patients with UAD. Thirdly, since our patients were volunteers who self-selected to be included into clinical trials, we could have been open to some sort of selection bias—in other words our data might not be representative of the wider asthma population in real life. Finally, we had no measures of allergic rhinitis such as total nasal symptom score or peak nasal inspiratory flow rate.

In summary, we have shown evidence to support the concept of unified allergic airway disease by demonstrating that in patients with allergic asthma, the presence of concomitant allergic rhinitis is associated with worse lung function and higher type 2 biomarkers. We therefore emphasise the importance of recognising the presence of concomitant allergic rhinitis and incorporating an algorithm combining type 2 biomarkers and lung function in assessing asthma patients.

## Data Availability

All data generated or analysed during this study are included in this published article (and its Additional files).

## Abbreviations

ACQ: asthma control questionnaire
CI: confidence interval
Eos: eosinophils
FEF_25–75_: forced expiratory flow at 25% to 75% of forced vital capacity
FeNO: fractional exhaled nitric oxide
FEV_1_: forced expiratory volume in 1 second
ICS: inhaled corticosteroids
INS: intranasal steroids
SPT: skin prick test
T2: type 2 airway inflammation
UAD: unified allergic airway disease
